# “Failing to Recognize me as a Fellow Human Being”: A Matrix of Dehumanization Structures Cancer Care for Trans and Nonbinary People With Cancer

**DOI:** 10.1002/pon.70648

**Published:** 2026-11-06

**Authors:** Ben C. D. Weideman, Rhea N. Alley, Lauren Love Pieczykolan, Allie Vanden Heuvel, B. R. Simon Rosser, G. Nic Rider

**Affiliations:** ^1^ Division of Epidemiology and Community Health University of Minnesota School of Public Health Minneapolis Minnesota USA; ^2^ National Center for Gender Health Eli Coleman Institute for Sexual and Gender Health Department of Family Medicine and Community Health University of Minnesota Medical School Minneapolis Minnesota USA

**Keywords:** cancer, dehumanization, intersectionality, nonbinary, oncology, transgender

## Abstract

**Background:**

Trans and nonbinary (TNB) people remain invisible in cancer care. Limited research indicates barriers to quality cancer treatment, including healthcare discrimination linked to poor survivorship outcomes.

**Aims:**

This study aimed to characterize cancer treatment experiences among TNB cancer patients using an intersectionality lens and exploring the structural processes underlying inequities.

**Methods:**

We recruited TNB cancer patients (*N* = 42) using multiple strategies and conducted individual, semi‐structured interviews (45–90 minutes). Interviews were transcribed by professional service and de‐identified. We used reflexive thematic analysis to generate themes from interview data.

**Results:**

We developed seven interconnected themes across three, mutually constitutive domains of power that compose a *matrix of dehumanization* in cancer care—a structural process that confers uneven risks of harm based on patients' multiple social positions. In the institutional systems domain, *The Climate We Live In* describes how anti‐trans politics are embedded in legal systems, and *A New Type of Consumer* explains how for‐profit cancer care exploits value from patients. In the cancer care organizations domain, *Made Invisible* articulates practices of erasure in clinics leading to unmet care needs, and *What Are You Doing Here?* elaborates hyper‐visibility and a lack of safety in clinic settings. In the socio‐structural processes domain, *Ingrained Racism* details racialized dehumanization in provider and staff interactions, *Losing Sexuality* captures sexual and gendered objectification, and *Continues to Hurt Me* highlights the enduring traumas of dehumanizing care.

**Conclusions:**

Improving cancer treatment inequities for TNB people requires disrupting the status quo power relations that sustain dehumanization in cancer care.

## 1 Introduction

Trans and nonbinary (TNB) people remain largely invisible in cancer care despite documented inequities across the cancer control continuum [1]. Available evidence points to elevated cancer risk and mortality in TNB communities due to HIV and HPV infection, stress, less screening, and other factors [2]. Yet, social stigma, barriers to research inclusion, and the absence of gender identity data collection by federal agencies suppress accurate reporting of TNB population size and cancer rates [3, 4]. The few studies focused on TNB cancer patients have highlighted major barriers to quality care [2], including healthcare discrimination linked to poor cancer survivorship outcomes [5]. Political efforts to restrict TNB people's access to healthcare have recently intensified, raising these barriers to cancer care [6]. Given that oppression and multi‐level stigma drive health inequities impacting TNB communities, now more than ever, it is crucial to examine these structural processes in healthcare [7].

Research on cancer treatment for TNB people is especially scarce [2]. In the 2022 U.S. Trans Survey, 47% of 92,329 TNB participants reported at least one past‐year negative healthcare experience (e.g., misgendering, harassment), mainly reflecting primary care [8]. The largest survey to date of TNB cancer patients (*n* = 211) did not measure these negative healthcare experiences [9]. Smaller surveys have documented cancer care discrimination, such as being dismissed and identity negation, but have sample sizes too limited to yield precise or generalizable prevalence estimates [10, 11]. The most illuminating evidence comes from select qualitative interview studies with TNB cancer patients. Interviews revealed poor treatment experiences, including transphobia and scarce affirming resources that heighten anxiety and care avoidance [12], gendered cancer clinics that alienate TNB people [13], stigmatizing provider assumptions about gender‐role and anatomy [14], and unmet sexual and reproductive care needs [15]. Apparent across these studies, cancer providers are often ill‐equipped to address the unique needs and impacts of cancer on TNB people due to a lack of gender‐affirming oncology knowledge and training [2].

Intersectionality is crucial yet underused framework for addressing cancer care inequities [16]. An intersectionality lens examines how interlocking systems of domination (e.g., white supremacy, capitalism, cisnormativity) create complex social inequities based on individuals' multiple intersecting identities [17]. Patricia Hill Collins conceptualizes this process as a “matrix of domination” functioning as a “structuring structure” that maintains inequities by shaping institutional systems like government and healthcare [18]. Despite critical emphasis on power, intersectionality in public health and medicine is often depoliticized and flattened to focus solely on individuals' identities [17]. The Intersectionality Research for Transgender Health Justice (IRTHJ) framework guides examination of TNB health inequities by centering power in analysis [19]. IRTHJ models how structures of domination are constituted through institutional systems and everyday socio‐structural processes (e.g., gendering, racializing) that (re)produce status quo power relations. IRTHJ also outlines three researcher actions for advancing TNB health justice: naming intersecting power relations, centering embodied knowledge, and disrupting the status quo. IRTHJ has not yet been applied to cancer.

Using an intersectionality lens, this study aimed to characterize cancer treatment experiences in, to our knowledge, the largest qualitative study to date of TNB cancer patients. During interviews, many participants named dehumanization as a defining feature of their cancer treatment. Dehumanization refers to a person's experience of being denied humanity, being seen as an object or something less than or even non‐human [20]. Psychologists developed the Pantheoretical Dehumanization Model to explain how intersecting oppressions activate dehumanization as an internal process (i.e., cognitive, psychological), contributing to mental health inequities in marginalized groups [20]. Although validated with TNB populations [21], the Pantheoretical model addresses only the internalization of dehumanization, not its production. This analysis offers a structural explanation behind TNB cancer treatment inequities called the *matrix of dehumanization* in cancer care.

## 2 Methods

We conducted individual interviews with TNB cancer patients as part of a larger project focused on treatment experiences and research recruitment methods among LGBTQ+ cancer patients [22]. This study was approved by the University of Minnesota Institutional Review Board (STUDY00016416). Between June 2023 and December 2024, we employed a previously described stratified purposive approach [4] to recruit three TNB subgroups: trans women, trans men, and nonbinary people. Forums included LGBTQ+ focused dating apps (e.g., HER), study recruitment sites, outreach at local events (e.g., elder lunches), and cancer support sites, with efforts to overrecruit participants of color.

Recruitment materials directed interested individuals to an online eligibility screener. Study inclusion criteria were having a cancer diagnosis, ≥ 18 years old, U.S. residency, and English proficiency. The screener also collected demographics and contact details. Eligible participants were contacted by telephone. We collected additional details about cancer type and treatment history. Individuals who had not undergone treatment were excluded. Those who remained eligible and interested completed informed consent and scheduled an interview.

Interviews were conducted remotely by telephone or Zoom (45–90 minutes). The semi‐structured interviewed guide explored participants' cancer treatment experiences with respect to their intersecting gender, sexual, and racial/ethnic identities, with questions about provider interactions, clinic practices, and discrimination (See Supporting Information S1: Appendix 1). Interviews were recorded and professionally transcribed, then verified for accuracy and redacted for identifying information. Participants were offered a $100 e‐gift card.

We used reflexive thematic analysis (RTA) to generate shared‐meaning‐based stories with interview data, with intersectionality as the interpretation framing theory [23]. RTA foregrounds researcher reflexivity as a source of rigor and encourages a flexible, “artfully interpretive,” and collaborative approach well‐suited to critically analyzing complex structural phenomena [23]. Braun et al.’s six‐step RTA process [23] guided our coding and theme generation for this paper (step‐by‐step details and reflexivity statement in Supporting Information S1: Appendix 2). This recursive process involved the team familiarizing themselves with the data, independently making preliminary code‐sets, and then collaboratively harmonizing them into a shared codebook. The team reiteratively applied the codebook to transcripts in Dedoose, meeting weekly to refine codes. The first author grouped coded data into core concepts, organizing them by adapting and extending the IRTHJ framework to develop initial themes. The team then refined themes and wrote cohesive narratives with illustrative quotes through a collective revision process. We agreed that information power was sufficient based on Malterud et al.’s criteria (Supporting Information S1: Appendix 2) [24]. We presented results and methodology following the RTA Reporting Guidelines (RTARG) [25].

## 3 Results

### 3.1 Participants

Interviews included 42 TNB participants (age range: 19–76 years) across three subgroups: trans women (*n* = 15), trans men (*n* = 8), and nonbinary people (*n* = 19; Table 1). Many gender identities were represented (e.g., genderqueer, transfeminine). Participants used many sexual identity descriptors (e.g., asexual, fluid, lesbian, queer). Just under half of the participants identified as exclusively White and Non‐Hispanic (48%). Racially marginalized identities included Black (36%), Hispanic/Latino/a/x/e (14%), American Indian (5%), and Asian (5%). The most common cancer types were lung (21%) and breast (17%). Time since most recent cancer diagnosis ranged 0.1–29 years (mean = 4.9; standard deviation: 5.8).

**TABLE 1:** Participant demographics (*N* = 42).

Characteristics	*n* (%)
Gender Subgroup	
Trans women	15 (36)
Trans men	8 (19)
Nonbinary people	19 (45)
Gender Assigned at Birth^a^	
Female	23 (55)
Male	19 (45)
Intersex	3 (7)
Gender Identity^a^	
Agender	2 (5)
Genderfluid	3 (7)
Genderqueer/queer	2 (5)
Man	8 (19)
Nonbinary	14 (38)
Transfeminine	1 (2)
Transmasculine	1 (2)
Woman	16 (38)
Two‐Spirit	
Two‐Spirit	2 (5)
Sexual Orientation^a^	
Aromantic	3 (7)
Asexual	3 (7)
Bisexual	12 (29)
Demisexual	2 (5)
Fluid	2 (5)
Gay	6 (14)
Lesbian	6 (14)
Pansexual	6 (14)
Polyamorous	3 (7)
Sapphic	1 (2)
Queer	11 (26)
Racial Identity^a^	
American indian	2 (5)
Asian	2 (5)
Black	15 (36)
White	25 (60)
Unknown	1 (2)
Ethnic Identity	
Hispanic or Latino/a/x/e	6 (14)
Age (years)	
18–29	11 (26)
30–39	15 (36)
40–49	11 (26)
50–59	3 (7)
60+	2 (5)
Cancer Types^a^	
Bone	2 (5)
Blood	2 (5)
Bladder	1 (2)
Breast	7 (17)
Cervical/ovarian	2 (5)
Leukemia	4 (10)
Lymphoma	4 (10)
Lung	9 (21)
Melanoma/skin	3 (7)
Prostate	4 (10)
Spinal	1 (2)
Testicular	2 (5)
Thyroid	4 (10)
Thymoma	1 (2)
Recruitment Method	
Cancer support sites	1 (2)
Dating apps	27 (64)
Local community events	2 (5)
Study recruitment sites	8 (19)
Referrals	4 (10)

^a^ Participants used multiple terms, so totals may not sum to 42.

### 3.2 Themes

Seven themes comprised the *matrix of dehumanization* in cancer care across three power domains (Figure 1): institutional systems, cancer care organizations, and socio‐structural processes. Additional supporting quotes are included in Table 2.

**FIGURE 1:**
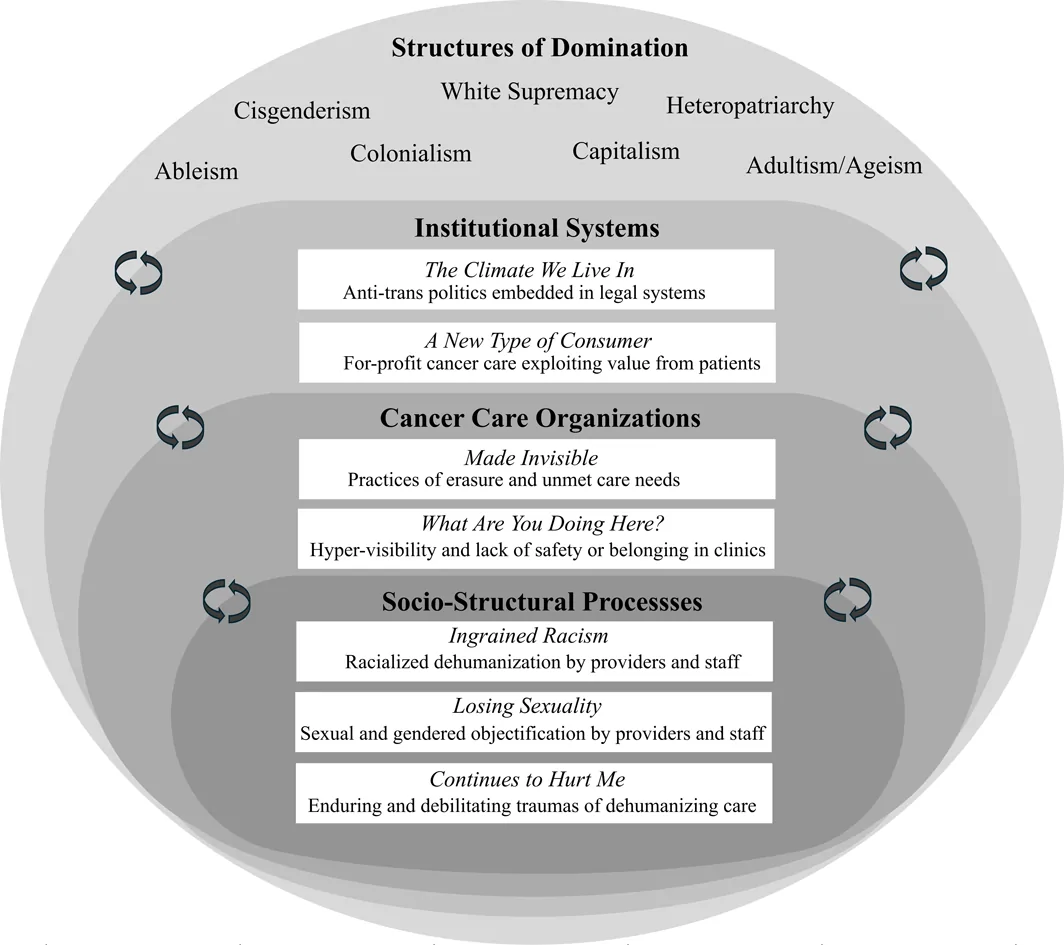
The matrix of dehumanization in cancer care.

**TABLE 2:** Additional supporting quotes.

Domain	Theme	Quotes
Institutional systems	The climate we live in	“Since I live in [conservative state], I'm kind of hesitant about [disclosing my identity at the clinic] because they're so viciously anti‐trans for no reason…picking on segments of people…to dehumanize them in order to use it as a strategy of getting votes that way, it's just stupid.” (*Trans Woman Subgroup, White, Leukemia*) “It was very difficult to get things like my name corrected in the [medical] system… trying to get records sent over to my new provider has been incredibly difficult because they're under a different name… I'm having to disclose my birth name to providers who have no reason to know it except for they have to get those records and connect them.” (*Nonbinary Subgroup, White, Osteosarcoma*) The treatments were getting rough and it got really hard for my mental sake because I would continuously hear my birth name and it gives me a lot of dysphoric feelings because obviously that's not me anymore. It was something I had to learn to cope with because obviously it's [the providers'] job. They're not doing it on purpose, they tried using my preferred name as much as they could. It was just whenever they had to do official things, they had to use my birth name because I wasn't able to change it and it was a little rough (*Trans Man, Middle Eastern, White, Leukemia*) “I feel like we are going to head back to the 1950s or worse where we are targeting people at this level. Or we are going to go back to things being illegal or people being punished or their kids…All of that is terrifying, honestly, as an individual…It makes me more fearful than I have been since I have been out.” (*Nonbinary Subgroup, White, Multiple Cancers*)
	A new type of consumer	“The business of cancer is better than curing cancer…The organizations that fundraise hundreds of millions of dollars [for cancer] and…do we really have anything to show for it? I mean, I see these things like…pink ribbon[s] and it all just feels like exploitable shit.” (*Nonbinary Subgroup, White, Spinal Cancer*) “Seeing breast cancer stuff that is completely gendered, pink…that makes me want to turn away…[and] sometimes it's something that's been recommended by my doctors…In advertising…all women who look a certain way, who all look very cis, heterosexual.” (*Nonbinary Subgroup, White, Breast Cancer*) “Cancer culture wants you to put on makeup and wigs and present yourself in a certain feminine way to hide and I wasn't willing to do that. So I walked around bald and didn't hide it and that made people feel very uncomfortable” (*Nonbinary Subgroup*, *Asian, Breast Cancer*) “I had a lot of decreased testosterone. And I had a lot of fatigue. And also it affected my intimate relationship with my partner. And also it was very expensive, the surgery. It was very expensive. A lot of insurance coverage was involved. That particular period when I had the surgery, I would say for straight 4 months, I was very moody and depressed because I was undergoing a lot of physical and health challenges in the development of my body, which the doctor said was also part of it. And also it had the potential effects in my workplace. Like, I couldn't go to work for 4 months blocks.” (*Trans Man Subgroup, Intersex, Black, Testicular Cancer*)
Cancer care organizations	Made invisible	“I would say that as far as being a queer body, there's no education on terminology, it's always ‘women’…I put genderfluid whenever there's an option; most of the time there isn't…it's male or female, that's it.” (*Nonbinary Subgroup, Two‐Spirit, American Indian, Cervical Cancer*) “I had to go into the AMAB [assigned male at birth] category [in the cancer study] because they had to take me off my estrogen…Detransitioning is hell…They put it as male and female [in the study]—that also kind of bothers me because it is putting a gender upon individuals” (*Trans Woman Subgroup, Intersex, White, Multiple Cancers*). “I'm seeing a specialist…I usually mark down on forms that I'm trans, but even if I mark it down, there's no effort to correct or anything. Sadly, I really don't expect them to.” (*Trans Woman Subgroup, White, Leukemia*) “I remember seeing a fair amount of general research studies…it's extremely prescriptive or using only biological terms like male or female, ignoring intersex. I know plenty of people that if it's gender male or female that's the only option they'll just stop taking surveys. Things that don't represent people, many people will stop engaging at that point. It's clear they are not welcome there.” (*Nonbinary Subgroup, White, Leukemia*) “I talked to my [cancer provider] about how I use injectable hormones, is there any issue with that? She was kind of taken aback…I don't feel like we're—trans people—are thought of in the process very well.” (*Trans Woman Subgroup, White, Lymphoma*)
	What are you doing here?	Apart from being multiple genders…the first thing that came to my mind when I entered the place was like, I'm a Black person.” (*Trans Man Subgroup, Intersex, Black, Testicular Cancer*) “With specific people there were sneers. Oh, there was a receptionist who looked at me and said ‘you're in the wrong place, honey,’ when I showed up for my radiation treatment…Childhood cancers are taken seriously, old age cancer taken seriously—20s to 35, not taken seriously. Then when you add in not presenting hyper femme, hyper masc, not fitting a gender role—not taken seriously” (*Trans Woman Subgroup, White, Multiple Cancers*) “[The Veteran's hospital is] definitely an organization oriented toward men…They struggle with pronouns and dealing with anybody other than cisgender [men].” (*Trans Woman Subgroup, White, Lymphoma*) “The breast cancer center is completely pink…they give you a pink gown to change into. It is completely gender coded…It's very much a space for women and I did feel like an outsider being in that space” (*N* *onbinary Subgroup, White, Breast Cance*r) “I'm not being treated by people who are like me.” (*Trans Woman Subgroup, White, Leukemia)*
Socio‐structural processes	Ingrained racism	“Just imagine a Black person being a trans man, the kind of racist comments, the hatred he…will be receiving these days…It was really hard for me…They should treat a patient like a human because we are human.” (*Trans Man Subgroup, Black, Breast Cancer*) “The one time I was burning with fever and I had to come to the hospital…I saw people of non‐color that came in and were given proper attention [immediately]…I was there for an hour, dying slowly. That was the worst treatment I've ever received in my life…I started thinking, as a human…how would they have treated you? Because they treated me like trash.” (*Trans Woman Subgroup, Black, Breast Cancer)* “I woke up from having emergency C‐section to a white doctor standing in my face accusing me of being a drug addict…. Even then, no one said I had elevated platelets. Even then, no one said let's see why she had this happen to her and the baby. Let's see why. No one thought to do that. I was just accused of being a drug addict…And so, that to me was one of the most horrific experiences that I had. It was just like on and on and on like me being a Black Indigenous woman, like nobody listened… and that's my first experience [of childbirth], you know, is being under the scrutiny of child protective services.” (*Nonbinary Subgroup, Black, Blood Cancer*)
	Losing sexuality	“I am very femme presenting, and my doctor was male…it's a little uncomfortable when an old cis guy kind of comes into the room and starts making comments about you when you identify as a they/them…But he's talking about you in a weird way. That's more of a female issue, but it still relates because I'm nonbinary. Just because I look female, doesn't mean I am female. And regardless, those comments are inappropriate. So I guess that's really the level of discrimination I've got—interestingly sexist comments.” (*Nonbinary Subgroup, White, Blood Cancer*) “I am also polyamorous…And depending on how cool the provider is, they will be like, ‘Oh, okay’ or I have had a couple of providers go, ‘Oh, are you cheating on someone,’….Or they're like, ‘Oh, player.’ I'm like, ‘no.’” (*Trans Woman Subgroup, White, Multiple Cancers*). “I did experience negative treatment in regard to sexism. I did have doctors who talked down to me.” (*Nonbinary Subgroup, White, Thyroid Cancer*) “I really wish that my concerns about my quality of life and the association with my sex life were met with understanding and awareness over downplaying and minimizing, invalidating of my experiences …[the] married, monogamous doctor [was] incredibly intrigued by my lifestyle…When he would ask me about my sexuality and my sexual practices, I could tell it was not normal for him…And some of the things he said were really odd, like, ‘…it'll be awhile before you all can get back to whatever it is you do regarding sex.’…He didn't seem uncomfortable, it seemed like more we were piqued curiosity for him…that felt disrespectful to me.” (*Nonbinary Subgroup, Two‐Spirit, American Indian, Cervical Cancer*)
	Continues to hurt Me	“They way they handled me…when I had IVs and things like that, it felt like they were rough and not caring.” (*Nonbinary Subgroup, Two‐Spirit, American Indian, Asian, Black, Ovarian Cancer*) “I did have doctors who would disregard my boundaries…I would tell the doctors straight up, please just tell me before you stick me [with the needle]…I'm in this incredibly vulnerable, physical position… and they would disregard it halfway through…So much of my formative years involved being manhandled by doctors, having needles in my throat, having all the tests done, having my body turned into a thing to manage.” (*Nonbinary Subgroup, White, Thyroid Cancer*) “I woke up from surgery…I was in excruciating pain… and it took a lot to get [the provider] to believe me that something was wrong… several visits later…he finally did an X‐ray and realized that he broke my shoulder blade… he still denied and tried to fight me the entire way saying he didn't do it…he finally admitted it… and to this day, it was still the most painful thing I've ever been through.” (*Nonbinary Subgroup, Black, Bone Cancer*) “The doctor was incredibly aggressive, she was demeaning toward me, she wants to do a biopsy and a curettage to see if they can really find the cancer….She's pulling my cervix down, and then she's getting in there with the curettage, it's painful as fuck. I wasn't prepared for it…She actually had pulled my pelvic—what do they call it—ligaments that hold up the uterus. She had pulled them so hard that I was in pain for 2 weeks afterward.” (*Nonbinary Subgroup, Two‐Spirit, American Indian, Cervical Cancer*)

#### 3.2.1 Institutional Systems

Institutional systems intersect in complex networks of laws, policies, and economies which enact structures of dominations [19], reflected in two themes (*The Climate We Live In; A New Type of Consumer*).

##### 3.2.1.1 The Climate We Live in

Participants characterized escalating anti‐TNB policies as political strategies of dehumanization, which increased stress and fear of discrimination in cancer care, as also reflected in other healthcare settings [26]. One participant explained how this eroded safety and deepened risky power asymmetries with providers:

Going to the doctors…feels like a gamble every time. It’s nerve wracking, it’s very scary, because half of them…don’t support hormone therapy and things like that… I’m not going to argue with a nurse because they’re the ones poking a needle in my arm…at least in the climate we live in.

(Tran Woman Subgroup, White, Leukemia)

The anti‐TNB socio‐political environment also exacerbated the entrenchment of the sex/gender binary in legal systems surrounding cancer treatment, which created care barriers and repeated institutional failures to recognize participants' personhood:

There was this consistent hassle of my insurance information still had my legal name and my driver’s license still had my legal name and I’m still clearly presenting as a woman but having to go in and talk to all the people…at the oncologist’s office…There’s always eight people that you deal with not counting the doctor. Of those, only the blood nurse and the checkout lady got [my name] right.

(Trans Woman Subgroup, White, Thymoma)

##### 3.2.1.2 A New Type of Consumer

Participants described for‐profit cancer care and surrounding economic systems as inherently objectifying. One participant summarized:

The second I became a cancer patient I was a new type of consumer…and all of that then amplifies those ideals of what you’re supposed to be as a woman and all of this… wigs, this and that. I was starting to question how much capitalism was framing my life.

(Nonbinary Subgroup, Asian, Breast Cancer)

This participant's cancer diagnosis subjected them to new markets that commercialized gendered products (e.g., “wigs”) in the pursuit of profit maximization. Another participant recalled the advertisements decorating walls where they underwent several surgeries to have “Melanoma carved off their face,” leaving them “traumatized”:

[In the clinic] there were a lot of…cutouts and stands of like ‘get the body you always wanted, be a better you’ and just like, you know, smiling white women eating their salads with the perfect body…And it’s just like…this is almost violently anti‐queer.

(Nonbinary Subgroup, Hispanic or Latino/a/x/e, Melanoma)

When this participant asked their dermatologist about foregoing reconstructive surgery due to trauma, they were told: “We can't do that…you really need to think of the reputation of the clinic that made you cancer free.”

These quotes emphasize that the capitalistic systems surrounding cancer care commodify gender, whiteness, and “anti‐queer” representations of health, simultaneously devaluing the personhood of TNB people and treating them as opportunities for value extraction. All the while financial debilitation from cancer was commonplace among participants (e.g., “I'm worried about if I can pay for care”), exemplifying research that indicates TNB cancer patients face a disproportionate burden of financial hardship and cost‐delayed care, particularly those who are also racially marginalized [27].

#### 3.2.2 Cancer Care Organizations

We added cancer care organizations as an important intermediary power domain governing social and material resource distribution [28], reflected in two themes (*Made Invisible*; *What Are You Doing Here?*).

##### 3.2.2.1 Made Invisible

Organizational policies and practices led to experiences of erasure. One participant described having the “most dysphoria after coming out” in the cancer clinic, partly because of the “long slog” of getting “gender changed in the system”:

After I started transitioning…they sent me to a male locker room to change. We had policies at the time. Even though I changed [the medical record]…nobody saw it. And I didn’t feel like I was in the place yet in my transitioning to say, ‘No, wait.’… I certainly didn’t belong in the men’s locker room—gender neutral would have been perfect. I wasn’t given that option. That was not a good feeling. I didn’t feel seen.

(Trans Woman Subgroup, White, Thyroid Cancer)

Several participants did not have an option to document their gender identity at all in their medical record, and some who participated in clinical research studies were similarly erased. As a result, many participants experienced unmet health care needs due to insufficiently knowledgeable providers, frequently documented in the literature [2, 14, 15]:

I felt like [medical staff] lacked training and awareness in how to interact with me. Also, it was stressful…[it] actually made me feel invisible.

(Trans Man Subgroup, Black, Lung Cancer)

Within cancer care, this theme reflects Bauer et al.’s foundational insight that institutional erasure (e.g., polices, documentation, infrastructure) and information erasure (e.g., information systems, training curricula) are mutually reinforcing and produce a pervasive cisnormativity in that renders TNB people invisible in healthcare [29].

##### 3.2.2.2 What Are You Doing Here?

This theme captured how TNB cancer patients unevenly experienced organizations built upon their erasure. Participants often felt hyper‐visible or like they did not belong in cancer clinics, particularly when their gender difference intersected with other minoritized identities, such as race or age. As also previously documented [13], participants explained that the gendered organization of clinics contributed to this alienation:

During the chemotherapy time…I would be mistaken for a boy. I was in a women’s cancer center and people would look at me and be like what are you doing here?…Very gendered… it felt like I wasn’t woman enough to be in there…The staff were all one demographic…It was like the 1950s, 40s where the women are doing all the office stuff and meandering around making sure everything is okay for the doctor who is a white man in an office led by a white woman. It’s somewhat a hostile environment especially if you’re not a straightforward white female who is straight…As soon as you walk in, for me at least, it was anxiety.

(Nonbinary Subgroup, Two‐Spirit, American Indian, Asian, Black, Ovarian Cancer)

This quote further illustrate how clinics are often designed such that TNB people have no place, while being staffed with predominantly white, cisgender healthcare professionals, often in highly gendered roles [30, 31]. The risk of being seen and targeted as outsiders within these places—and thus dehumanized—varied according to participants' intersecting social positions, with multiply marginalized participants (e.g., TNB people of color) facing the greatest harm.

#### 3.2.3 Socio‐Structural Processes

Dehumanizing interactions with providers were socio‐structural processes that (re)produced status quo power relations across three themes (*Ingrained Racism*; *Losing Sexuality*; *Continues to Hurt Me*).

##### 3.2.3.1 Ingrained Racism

Participants of color reported especially intense experiences of dehumanizing treatment:

[The nurses] were a little bit racist…Some of them were a little bit harsh to me…not giving me much attention…They were failing to recognize me as a fellow human being. Being trans, some of them think that you are no longer a human, you’ve done something that is [in] a way inhuman.

(Trans Woman Subgroup, Black, Prostate Cancer)

These dehumanizing interactions were not solely interpersonal but connected to broader racist structures and histories that shaped their gendered experiences as well, as another participant explained:

[I] come in appearing like a tall, Black boy. Therein, I am intimidating to them because I’m not a white female and what they’re expecting…[Providers] have training and bedside manner and things like that but they really only pertain that to people who they deem human. And I feel like, for me, it’s that ingrained racism that they were raised with…[The clinic is in] a town known for their hangings…they have very few people of color of any type, be it Black, Hispanic, Asian, anything other than non‐melanated skin tones and European descent who have claimed America. You’re just not welcome there…You can really see the care that they have for the other people who they deemed humans. I guess that’s my point…When people don’t look at you as human, then it’s down from there.

(Nonbinary Subgroup, Two‐Spirit, American Indian, Asian, Black, Ovarian Cancer)

This theme reflects that racism is a fundamental driver of health inequities, manifesting across the cancer care continuum [32], and driving disproportionate healthcare victimization and abuse by providers among TNB people of color [33].

##### 3.2.3.2 Losing Sexuality

Participants described how providers and staff interactions often made their sexual and gendered bodies feel like objects, taking away agency, autonomy, and personhood. One participant explained:

In radiation, you’re exposed, your chest is exposed to a room full of people. After a while, you just are a medical specimen. You lose a sense of your sexuality. It’s a violence that happens to you. It’s dehumanizing, and I ended up disassociating a lot. It was very traumatic.

(Nonbinary Subgroup, Asian, Breast Cancer)

Additional participants discussed that they felt de‐sexualized, hyper‐sexualized, and experienced sexist comments. These dimensions of sexual objectification reveal additional ways that provider behavior reinforces gender norms in cancer care [13, 14, 15]. When this influenced care decisions, it could cause lasting damage:

I knew I very much wanted…to [have] my testicles removed…but I was kind of forcibly given a prosthesis, some fake testicles, which I didn’t particularly want, but they were kind of forced on me. That certainly left a mental scar and physical baggage for me since then….My surgeon [who] did the orchiectomy was also a dude, and there was definitely some backslapping. ‘Oh boy, I just feel so bad for ya, buddy’.

(Trans Woman Subgroup, White, Lymphoma)

##### 3.2.3.3 Continues to Hurt Me

Several participants experienced rough treatment, boundary violations, and ensuing physical and psychological traumas at the hands of providers. One participant connected this dehumanizing care to the confluence of their marginalized identities:

When I told [the provider that the biopsy] was really painful, he’s like we’re almost done and didn’t do anything about it. But then proceeded to continue to hurt me and I feel like for me that was a real traumatic experience that reminded me what I already know about the ways that I move throughout the world and how people perceive me and how that affects the way that they treat me in my humanity or not… Doctors don’t know what to do with me…I’m a human being, I’m not a specimen…I think that a lot of it is because of who I am. It’s because I am queer, nonbinary, Latinx person of color that is coming before them and they’ve never seen a patient like me.

(Nonbinary Subgroup, Hispanic or Latino/a/x/e, Breast Cancer)

Traumatic interactions with providers were not isolated, individual incidents but a crucible where histories of structural dehumanization converge on a person placed in a precarious position by those very histories. These moments *continue to hurt* by echoing and amplifying structural harms.

## 4 Discussion

In this qualitative study, we applied an intersectionality lens to surface deep and multidimensional experiences of dehumanization in cancer care by centering the embodied knowledge of TNB cancer patients. Seven themes conveyed the dynamic workings of a *matrix of dehumanization* comprised of institutional systems, organizations, and socio‐structural processes that confer uneven risks based on patients' multiple social positions. This matrix operates as a system: each theme within and across each power domain mutually constitutes one another. Consistent with the Pantheoretical Dehumanization framework [20, 21], the dehumanizing treatment experiences shared by participants—involving stress, unmet care needs, financial devastation, discrimination, sexual objectification, and enduring physical and psychological traumas—are manifestations of broader structures of domination which most acutely harm multiply marginalized people. Our model provides a theoretical foundation for advancing intersectionality research and interventions to name and disrupt the status quo power relations (re)producing TNB cancer care inequities.

### 4.1 Implications

Researchers must consider how political factors in institutional systems shape TNB cancer care inequities. Previous research links restrictive U.S. state policies to a greater cancer burden and poorer physical and cognitive health among LGBTQ+ cancer survivors, with treatment inequities as a possible mechanism [34]. After years of rising anti‐TNB policies, recent federal actions (e.g., executive orders establishing binary sex definitions, spreading disinformation, and defunding TNB research) likely accelerate TNB erasure and dehumanization in cancer care [6]. These policies also perpetuate exclusionary data collection practices and chronic underinvestment in TNB cancer research [3, 35]. *The Climate We Live In* suggests these policies compound negative treatment experiences, and previous work indicates that both exacerbate health system mistrust and reduce research participation among TNB cancer patients, particularly TNB people of color [4]. These structural barriers sustain a limited clinical‐evidence base that leaves providers ill‐equipped to address TNB cancer patients' gender embodiment, dysphoria and euphoria, and sexual and reproductive health [2, 15, 36], which contribute to unmet care needs and dehumanizing encounters apparent in our results.

Researchers should also examine how economic factors contribute to TNB cancer treatment inequities. *A New Type of Consumer* emphasized how profit‐driven cancer care extracted value from TNB people while simultaneously marketing “violently anti‐queer” cultural representations of cancer patients (e.g., white, thin, cisgender), which may shape access to care and resources. For example, facing disproportionate financial toxicity from cancer [27], TNB cancer patients often turn to crowdfunding to afford treatment [37]. Yet, they raise less fund than their cisgender peers, particularly in anti‐TNB policy states [37]. Research suggests crowdfunding disparities may reflect TNB people's difficulty marketing themselves to a largely cisgender, white, and affluent donor base when they do not meet such dominant cultural representations [38]. Financial insecurity likely deepens unsafe power asymmetries with cancer providers, limiting TNB patients' ability to advocate for their needs or seek alternative care. Cultural representations of deservingness may also contribute to harmful provider behaviors, including the enforcement of gender assumptions when provisioning care, as observed in our results and prior research [14]. This dynamic may be worsened by evidential uncertainty stemming from a lack of TNB cancer treatment protocols [39], warranting further investigation.

Our *matrix of dehumanization* model extends the IRTHJ framework by introducing organizations as an important and undertheorized [28] power domain in the production of TNB health inequities. Results indicated that recognition of TNB cancer patients' humanity often hangs in the balance of routine organizational decisions shaping resource allocations. *Made Invisible* and *What Are You Doing Here?* converge in showing how cancer clinics are built upon the erasure of TNB people while simultaneously making TNB cancer patients hyper‐visible and unsafe in these settings. This cycle also differentially and systematically excludes TNB cancer patients from information systems and clinic‐based research based on their multiple social positions [4, 29], perpetuating erasure and dehumanization, particularly for those multiply marginalized. Cathcart‐Rake et al. recently published organizational strategies for gender‐affirming oncology practices (e.g., inclusive documentation, gender‐neutral facilities, and affirming imagery) [40], which offer a starting point for changing cancer clinic policies and practices to advance TNB cancer health equity.

A key conclusion is that dehumanizing interactions with cancer providers—described in *Ingrained Racism, Losing Sexuality,* and *Continues to Hurt Me—*were crystallizations of multidimensional structures of oppression. This was especially evident among racially marginalized TNB cancer patients, who experienced intense, racialized dehumanization, and remain understudied. Our model emphasizes that these dehumanizing encounters enable inequitable organizations and institutions to persist, thereby (re)producing dehumanization in cancer care. Bybee et al. recently published useful evidence‐based recommendations for improving TNB cancer care, including provider education, increasing TNB healthcare staff, and partnering with queer community organizations [6]. We caution that interventions focused solely on individual behavior change, generalized non‐discrimination, or one‐size‐fits‐all approaches will be insufficient to address TNB patients' unique needs and the structural forces that dehumanize them. Provider education should encourage systems‐level thinking, drawing attention to systems described here and equipping clinicians to advocate for justice in clinic and institutional policies and practices. Future intersectionality research in this area should examine specific intersecting dimensions of marginalization (e.g., disability and class) that were not adequately explored in this study to further elucidate how oppression functions in cancer care. Additionally, strengths‐based, resilience‐, and resistance‐oriented frameworks should be used to examine how TNB cancer patients and support communities, including healthcare allies, navigate and challenge dehumanizing structures, guiding efforts to disrupt them.

### 4.2 Limitations

This study was limited by partial perspectives in our participant pool and data generation process. We did not focus on differences by gender, sexual orientation, or cancer type, which may obscure important within‐group variation. The interview guide asked specific questions related to race, sexuality, and gender, and we did not consistently ask for experiences related to other categories of power such as ability or class. Over half our participants were young adults (< 40 years), with older patients less represented. Although our participants were more racially diverse than previous qualitative studies [12, 13, 14, 15], only a few individuals represented some subgroups (e.g., American Indian). Therefore, our findings on racism may not capture how it operates in distinct and nuanced ways across racial/ethnic identities.

## 5 Conclusion

We undertook this paper to understand the power relations that rendered dehumanization a defining feature of cancer care, as described by TNB cancer patients. By centering their embodied knowledge, we began to excavate a matrix of dehumanization in cancer care. This work further developed the concept of dehumanization as a structural process, deepening understanding of how healthcare is imbricated in broader systems of oppression. Participant insights reveal critical pathways for disrupting the power hierarchies that inhibit social and health equity for all [19]. Improving cancer care for TNB cancer patients requires upending the status quo, moving beyond individual interventions, and advancing intersectional change. One participant put it well:

You can really see the care that [providers] have for the other people who they deemed humans. I guess that’s my point… When people don’t look at you as human, then it’s down from there.

## Author Contributions

**Ben C. D. Weideman:** conceptualization, methodology, formal analysis, investigation, data curation, writing – original draft, writing – review and editing, visualization. **Rhea N. Alley:** conceptualization, methodology, formal analysis, investigation, data curation, writing – review and editing. **Lauren Love Pieczykolan:** conceptualization, methodology, formal analysis, investigation, data curation, writing – review and editing. **Allie Vanden Heuvel:** conceptualization, methodology, formal analysis, investigation, data curation, writing – review and editing, project administration. **B. R. Simon Rosser:** conceptualization, methodology, resources, writing – review and editing, supervision, funding acquisition. **G. Nic Rider:** conceptualization, methodology, formal analysis, investigation, resources, writing – review and editing, supervision, funding acquisition.

## Conflicts of Interest

The authors declare no conflicts of interest.

## Supporting information


Suppporting Information S1

Suppporting Information S2

## Data Availability

The authors have nothing to report.
