# Supplementary material for: “Failing to Recognize me as a Fellow Human Being”: A Matrix of Dehumanization Structures Cancer Care for Trans and Nonbinary People With Cancer
Source: Psychooncology. 2026 Nov 6;35(11):e70648. doi: 10.1002/pon.70648 (PMC13743854; doi:10.1002/pon.70648)
Supplement: Supplementary file 1 [file PON-35-e70648-s001.docx]

**Appendix 1: Semi-structured Interview Guide**

To give me a better sense of your experience, I’ll start by asking:

Can you tell me a little bit about how you found the Elsie Study and what motivated you to join?

*Prompt:* Was there something in the images or words that really caught your attention?

1. How do you describe yourself in terms of your sexual orientation? And how about your gender?

a. *Prompt:* What are some more identities that are important to you?
b. *Prompt (if needed):* How do you describe yourself in terms of your race?

**Part I: Experiences in Treatment**

Next, I’d like to ask you about your experience in being treated for cancer, especially as someone who is <<*fill in participant’s identity label(s)>>.*

2. Please tell me about your *overall* experience in cancer treatment.

3. Were you comfortable sharing that you are <<*fill in participant’s identity label(s)*>> to your healthcare providers?

a. *Prompt:* During your cancer treatment, did your healthcare providers speak to your specific needs as a <*fill in participants identity label(s)*>> person?
   i. If yes, what needs and how so?
   ii. If no, what do you wish they would have talked with you about?
b. *Prompt:* In the context of cancer care, did you feel that you received good information about your sexual health? How about health information related to your sexual orientation and gender?

4. *For White Non-Hispanic/Latino LGBTQ+ People:* How LGBTQ+ friendly and accessible was the place you were treated at?

*For LGBTQ+ People of Color:* How friendly and accessible was the place you were treated at for LGBTQ+ people of color?

a. *Prompt:* What made it so?
b. *Prompt:* What was missing from that place you were treated at that could have made it better?

5. Did you experience any discrimination, harassment, negative experiences, or poor treatment in the care that you received?

a. *Probe:* Why do you think that happened?
b. *Prompt:* Did your experience(s) change your willingness to participate in cancer research studies? What do you think?

6. Some people can feel stigmatized as a person with cancer. By stigma we mean that family, friends or others look at you or treat you differently because you have cancer. Please tell me your experience as a <<insert identity label(s) here >> person with cancer.

a. *Prompt:* What spaces or communities have you found the most support in your cancer experience?
b. *Prompt:* Was your experience in LGBTQ+ spaces different after sharing about having cancer?

**Part II: Participation in Cancer Research**

I’m going to switch gears now and ask you more about your experience and thoughts on participating in cancer studies.

7. Have you ever participated in a cancer research study (apart from this one)?

a. *Follow-up*: How many cancer studies have you participated in?
b. *Prompt:* What was that experience like?

8. How do you feel about participating in cancer research?

a. *Prompt:* If the study were funded by the federal government or say the National Institutes of Health, would that change how you feel about participating in cancer research?
b. *Prompt*: What concerns, if any, do you have about how your information will be used?
c. *Prompt:* What would you need to feel safer to participate in cancer research?

9. What are some barriers that *<<fill in appropriate label(s)>>* people might face if they participate in cancer research?

a. *Prompt:* What about any issues with discrimination? Is that a concern?

10. And what do you think would help other <<*fill in appropriate label(s)*>> people participate in cancer research studies?

a. *Prompt:* Have you heard of other <<*fill in appropriate label(s)*>> people that have had a good experience in cancer research? What would you be willing to share about those experiences?
b. *Prompt:* What are some of the factors that make you trust cancer research? What are some of the factors that make you not trust cancer research?

11. What are some things that would help build trust in your communities to increase participation in cancer research?

**Part III: Advertising**

Finally, I’d like to talk about research studies that could happen in the future, and what researchers could consider when they plan studies. We are going to talk about places that we can recruit from (such as places where people are treated for cancer, community organizations, online sites) that are trusted.

12. How would you feel if you were invited to participate in cancer research from your place of treatment?

a. *Prompt*: How would you feel about participating in a study recommended by the place where you received your cancer treatment?
b. How would you feel about your electronic health records being used as a way to identify you and recruit for research studies? Let’s say a researcher calls or emails people from a list whose electronic health records show they identify as a certain sexual orientation or gender identity with a cancer diagnosis, and they are already listed in their health record as being open to participating in research.

13. Are there any community organizations we should partner with (or maybe not partner with) which would influence your willingness to participate?

14. In terms of online recruitment, what specific sites do you use where you might respond to an advertisement? Let’s start with:

i. Cancer support sites?
ii. Social media sites?
iii. Dating sites/apps?

15. Out of these three different places that we have just discussed (places for cancer treatment, community organizations, online sites), which one would be the most likely for you to respond to?

a. *Prompt:* Do you feel differently about seeing an advertisement at a place where you are treated at compared with community organizations? Compared with online sites?

16. Do you have other ideas you’d recommend to help us recruit <<*insert identity label(s) here*>> people/communities who have been diagnosed with cancer?

17. Would compensation impact your decision to participate in cancer research?

18. Before we finish the interview, I’d like to ask you if you have any additional comments on making cancer research more friendly and accessible?

19. Lastly, would you like us to send you a summary of these study results when complete? And would you have interest in potentially being contacted about opportunities to be involved in future studies similar to this?
