# Supplementary material for: “Failing to Recognize me as a Fellow Human Being”: A Matrix of Dehumanization Structures Cancer Care for Trans and Nonbinary People With Cancer
Source: Psychooncology. 2026 Nov 6;35(11):e70648. doi: 10.1002/pon.70648 (PMC13743854; doi:10.1002/pon.70648)
Supplement: Supplementary file 2 [file PON-35-e70648-s002.docx]

**Appendix 2:** Reflexive Thematic Analysis Process

We analyzed qualitative interview data using reflexive thematic analysis (RTA), with intersectionality as a framing theory. The analyst team met weekly and worked independently during each phase of RTA, guided by Braun et al.’s (2022) six-step process:^1^

*1) Familiarization*. Each team member reviewed all transcripts and were assigned a specific subgroup for deeper immersion through re-reading, note-taking, and reflexive journaling.

*2) Coding*. Each team member independently created a preliminary set of codes from salient features of the data in their assigned subgroup. The team harmonized these code sets into a single codebook with shared definitions. The team then met weekly to raise interpretive questions, revise codes, and refine definitions as the codebook was reiteratively applied to each transcript using Dedoose qualitative software. Multiple team members reviewed each coded transcript, noting questions and disagreements which were resolved by the group during the weekly meetings. During the coding process, the team noticed the recurring and intense experiences of dehumanization described by TNB participants, which formed the basis for an overall story that became the focus of this paper and guided initial theme generation.

*3) Initial theme generation.* The first author first adopted an “experientially oriented” approach^1^ to identify and group coded data related to this article’s aim. This involved collating quotes and clustering codes into core concepts within the overarching narrative of dehumanization, using an intersectionality lens to frame interpretation. This approach strove to be organic, empathetic, and to emphasize participants’ embodied knowledge and how they made sense of their worlds.

*4) Developing and reviewing themes.* The first author then adopted a “critically oriented” approach,^1^ using the IRTHJ framework^2^ to organize and develop initial themes. This analytic process adapted and extended IRTHJ to theorize how themes interconnect, as well as recursively revised themes and produced written narratives using extensive participant quotes. The first author also created a preliminary model, based on the IRTHJ framework, to visualize how themes fit together, with short definitions for each. We drew on Collins concept of a “matrix of domination”^3^ when naming the complete *matrix of dehumanization* model to both reference the intersectionality lens used for analysis and theme generation, and to draw together a cohesive story across themes consistent with RTA.

*5) Defining, refining, and naming themes.* The team collaboratively reviewed the written narratives, selected illustrative quotes, settled on final names and definitions, and honed each theme within and across the model. The team also provided feedback on the model’s visual construction, which the first author incorporated and revised accordingly. We assessed information power of themes using Malterud et al.’s (2016) criteria.^4^

*6) Producing the report.* The team finalized cohesive narratives for each theme with representative quotes through a process of collective revision. This went through several stages of revision during the peer-review and publication process.

*Information Power Assessment*

| **Malterud’s Facets of Information Power** | **Information Power in this Study** |
| --- | --- |
| Aim – broad or narrow? | Broad exploration* of structuration of cancer care for trans and nonbinary adult cancer patients but with narrow focus on cancer treatment experiences |
| Sample specificity – dense or sparse? | Dense: trans and nonbinary adult cancer patients who received cancer treatment |
| Established theory – applied or not? | Applied theory: Intersectionality Research for Transgender Health Justice Framework^2^ |
| Dialogue quality – weak or strong? | Strong: semi-structured interview guide; open questioning; practiced interviewers; queer, trans, and nonbinary researchers and staff |
| Analysis strategy – case or across case? | Across case* analysis exploring a range of cancer treatment experiences |

* Malterud et al.^4^ suggest that these requires larger participant pools/data generation.

Our interviews and analytic approach used established theory to broadly explore cancer treatment experiences across cases of trans and nonbinary cancer patients with dense sample specificity and strong dialogue quality. Previous interview studies with trans and nonbinary adult cancer patients had sample sizes ranging from 7 to 17 participants.^5-7^ Based on Malterud et al.’s criteria, the team agreed that our 42-participant interview study provided the information power for valuable insight into our research question.

*Reflexivity Statement*

We engaged in processes of reflexivity throughout the data collection and analysis. In principle, this required critical reflection about how our social and cultural backgrounds, assumptions, positionalities, and power relations within the research team and institutional setting influenced the research process, such as interactions with participants. In practice, these reflections occurred as points of discussion and decision-making during weekly meetings, reflexive journaling about interviews, memo-writing in Dedoose during coding, and co-interpretation and writing of results. These served as opportunities to attend to how the analyst team’s various social identities and positions inevitably shaped data collection and interpretation of participant experiences. Our team included trans, nonbinary, and cisgender individuals, and people with diverse gender identities, sexual identities, racial/ethnic identities, training levels, and ages. The team also included people who had received cancer treatment themselves or had experienced cancer caregiving.

**References**

1. Braun V, Clarke V, Hayfield N, Davey L, Jenkinson E. Doing Reflexive Thematic Analysis. In: Bager-Charleson S, McBeath A, eds. *Supporting Research in Counselling and Psychotherapy: Qualitative, Quantitative, and Mixed Methods Research*. Palgrave Macmillan; 2022.

2. Wesp LM, Malcoe LH, Elliott A, Poteat T. Intersectionality Research for Transgender Health Justice: A Theory-Driven Conceptual Framework for Structural Analysis of Transgender Health Inequities. *Transgender Health*. 2019;4(1):287-296.

3. Collins PH. The Difference that Power Makes: Intersectionality and Participatory Democracy. *Investigaciones Feministas*. 2017;8(1):19-39.

4. Malterud K, Siersma VD, Guassora AD. Sample Size in Qualitative Interview Studies: Guided by Information Power. *Qual Health Res*. Nov 2016;26(13):1753-1760. doi:10.1177/1049732315617444

5. Lisy K, Kerr L, Jefford M, Fisher C. "Everything's a fight": A qualitative study of the cancer survivorship experiences of transgender and gender diverse Australians. *Cancer Med*. Jun 2023;12(11):12739-12748. doi:10.1002/cam4.5906

6. Kerr L, Fisher CM, Jones T. "I'm Not From Another Planet": The Alienating Cancer Care Experiences of Trans and Gender-Diverse People. *Cancer Nurs*. 2021 Nov-Dec 01 2021;44(6):E438-E446. doi:10.1097/NCC.0000000000000857

7. Alpert AB, Gampa V, Lytle MC, et al. I'm not putting on that floral gown: Enforcement and resistance of gender expectations for transgender people with cancer. *Patient Educ Couns*. Oct 2021;104(10):2552-2558. doi:10.1016/j.pec.2021.03.007

7. Gorman JR, Corey SL, Ginavan I, Garcia J. "Just for pregnant women, not for you": a qualitative evaluation of the sexual and reproductive healthcare experiences of transgender and gender diverse cancer survivors. *J Cancer Surviv*. Jul 09 2024;doi:10.1007/s11764-024-01633-z
